# Real-world patient characteristics associated with survival of 2 years or more after radium-223 treatment for metastatic castration-resistant prostate cancer (EPIX study)

**DOI:** 10.1038/s41391-021-00488-0

**Published:** 2022-02-21

**Authors:** Daniel J. George, Neeraj Agarwal, Oliver Sartor, Cora N. Sternberg, Bertrand Tombal, Fred Saad, Kurt Miller, Niculae Constantinovici, Helen Guo, John Reeves, XiaoLong Jiao, Per Sandström, Frank Verholen, Celestia S. Higano, Neal Shore

**Affiliations:** 1 Departments of Medicine and Surgery, Duke Cancer Institute, Duke University, Durham, NC USA; 2 Huntsman Cancer Institute, University of Utah, Salt Lake City, UT USA; 3 Tulane Cancer Center, Tulane University School of Medicine, New Orleans, LA USA; 4 Englander Institute for Precision Medicine, Weill Cornell Department of Medicine, Meyer Cancer Center, New York-Presbyterian Hospital, New York, NY USA; 5 Division of Urology, IREC, Cliniques Universitaires Saint-Luc, UC Louvain, Brussels, Belgium; 6 University of Montreal Hospital Center, Montreal, QC Canada; 7 Charité – Universitätsmedizin Berlin, Urologische Klinik und Hochschulambulanz, Berlin, Germany; 8 Bayer Consumer Care, Basel, Switzerland; 9 Bayer HealthCare Pharmaceuticals, Whippany, NJ USA; 10 Department of Medicine, University of Washington and Fred Hutchinson Cancer Research Center, Seattle, WA USA; 11 Carolina Urologic Research Center, Myrtle Beach, SC USA

**Keywords:** Prostate cancer, Prostate cancer

## Abstract

**Background:**

The real-world EPIX study was conducted to gather information about the characteristics of patients with metastatic castration-resistant prostate cancer (mCRPC) who survived ≥2 years after treatment with the alpha-emitter radium-223.

**Methods:**

This retrospective study of electronic health records in the US Flatiron database (NCT04516161) included patients with mCRPC treated with radium-223 between January 2013 and June 2019. Median overall survival (OS) and prostate-specific antigen (PSA) response (≥50% reduction) from start of radium-223 treatment were the primary and secondary endpoints, respectively. Patient characteristics were compared between those who survived ≥2 years versus <2 years, including a subgroup who survived <6 months.

**Results:**

In the 1180 patients identified, median OS was 12.9 months (95% CI: 12.1–13.7), and 13% of patients with data at 6 months had a PSA response. The survival groups included 775 patients (65.7%) who survived <2 years (including 264 (22.4%) who survived <6 months) and 185 patients (15.7%) who survived ≥2 years; 220 patients (18.6%) had incomplete follow-up data and were censored. On multivariate analysis, age >75 years, Eastern Cooperative Oncology Group performance status (ECOG PS) 2–4, visceral metastases, prior symptomatic skeletal events (SSEs), and prior chemotherapy were independently prognostic of reduced OS. For patients with survival ≥2 years versus <2 years, median age was 71 versus 75 years, 4% versus 14% had ECOG PS 2–4, 4% versus 10% had visceral metastases, 38% versus 44% had prior SSEs, and 16% versus 32% had prior chemotherapy.

**Conclusions:**

In this study of men with mCRPC treated in real-world clinical practice, median OS was consistent with that seen in the phase 3 ALSYMPCA trial. Patients who survived ≥2 years after the start of radium-223 were younger and had better ECOG PS, lower disease burden, and less use of prior chemotherapy than those who survived <2 years.

## Introduction

Since the pivotal phase 3 trial of the alpha emitter radium-223 (ALSYMPCA [1]), the mCRPC treatment landscape has evolved. Novel hormone agents were not available outside clinical trials during ALSYMPCA, but are widely available now, and chemotherapy is typically delayed to later lines [2]. In clinical practice, radium-223 is used in various lines of therapy, either as monotherapy or in combination with other agents, and is frequently administered following or layered onto sequential novel hormone agents [2]. Radium-223 may also be used before chemotherapy: in a subgroup analysis of participants enrolled in ALSYMPCA who had not previously received chemotherapy, treatment with radium-223 prolonged median overall survival (OS) by 4.6 months compared with placebo (16.1 vs 11.5 months, respectively, HR 0.69, 95% CI: 0.52–0.92; *p* = 0.01) [3].

Choosing the appropriate patient and treatment sequence is important to optimize the survival benefit from radium-223 [4]. The present real-world study was conducted to help identify patients with mCRPC in whom radium-223 may offer the most benefit, by assessing the characteristics of patients who survived ≥2 versus <2 years after the start of radium-223 therapy.

## Patients and methods

### Study design and patients

This retrospective cohort study used data from an electronic health record (EHR)-derived database. Flatiron (https://flatiron.com/real-world-evidence/) is a longitudinal, demographically and geographically varied database comprising EHR data from >280 community clinics and academic institutions, with data from almost 3 million patients with cancer in the USA available for analysis. The present cohort study included adult patients (aged ≥18 years at diagnosis) with mCRPC treated with radium-223 as monotherapy or in combination with other life-prolonging anticancer therapies (e.g., abiraterone acetate and prednisone; enzalutamide; and/or taxanes) between January 2013 and June 2019. A cut-off date of December 2019 was applied for the follow-up data. Patients involved in clinical trials were excluded.

### Study endpoints and analyses

The primary outcome measure was median OS from start of radium-223 therapy. Kaplan–Meier estimates of OS were calculated for the overall cohort, with multivariate Cox proportional hazards models used to identify significant predictors of OS. Patients alive at the time of database cut-off were censored at the last date known to be alive. SAS version 9.4 (SAS Institute Inc., Cary, NC, USA) was used for statistical analyses.

PSA response, defined as a 50% or greater reduction in baseline PSA level after initiation of radium-223, was the secondary endpoint, measured in patients with baseline PSA ≥10 μg/L and with ≥1 PSA measurement between 15 days and 6 months after radium-223 start. Alkaline phosphatase (ALP) response, defined as a ≥30% reduction in ALP from baseline at any timepoint after initiation of radium-223 therapy, was also assessed in patients who had ALP levels measured at baseline and at least once >15 days after starting radium-223 therapy.

Baseline characteristics and treatments were reported descriptively for the overall cohort and for patients with survival ≥2 years and <2 years (including a subgroup with survival <6 months). Categorical variables were measured as frequencies and percentages, whereas mean (standard deviation [SD]) or median (range) was used for continuous variables.

### Study ethics

As this study was a retrospective review of EHR data, no Institutional Review Board review or patient informed consent was required.

## Results

### Patient characteristics

**Fig. 1:**
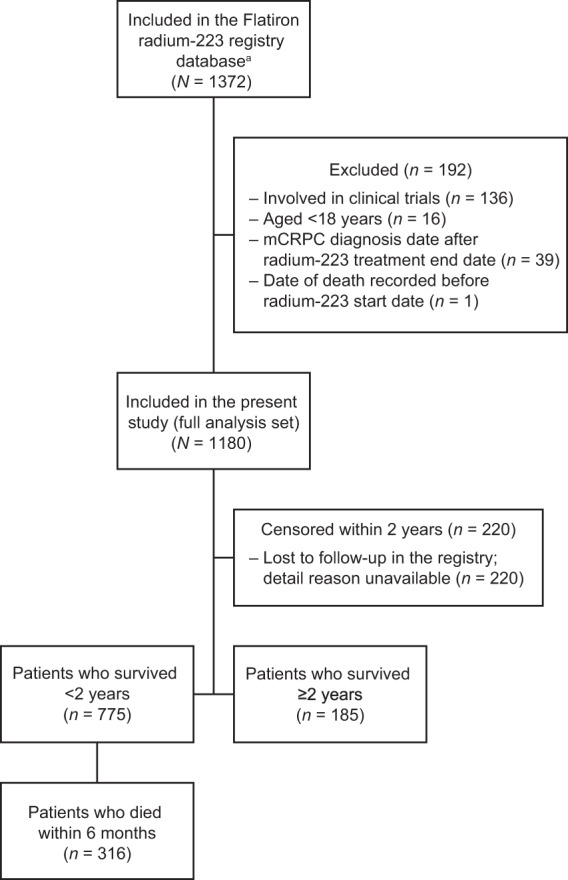
Patient flow diagram. ^a^Patients with metastatic castration-resistant prostate cancer treated with radium-223 any time between January 2013 and June 2019.

The study included 1180 patients with mCRPC from the Flatiron database who received radium-223 between January 2013 and June 2019 (full analysis set; Fig. 1). The group with overall survival <2 years after radium-223 comprised 775 patients (including 264 patients with survival <6 months), and the group with survival ≥2 years after radium-223 comprised 185 patients; 220 patients were censored within 2 years because of insufficient follow-up data and were excluded from the descriptive group analyses.

Baseline patient and disease characteristics for all patients and by survival duration are shown in Table 1. For patients who survived ≥2 years versus <2 years, median age was slightly lower (71 vs 74 years); median time from mCRPC diagnosis to the start of radium-223 therapy was shorter (7.1 vs 9.9 months); the proportion of patients with bone-only metastases was greater (86% vs 79%), and the incidence of prior symptomatic skeletal events (SSEs) was lower (38% vs 44%). In addition, patients who survived ≥2 years versus <2 years had lower median levels of ALP (78 vs 133 U/L) and lactate dehydrogenase (LDH; 188.5 vs 227.5 U/L) at baseline, although only 226 patients had a documented baseline value for LDH. Baseline median hemoglobin level in the group with survival ≥2 years (12.8 g/dL) was close to the normal range (≥13.0 g/dL in men [5]) and greater than in the group with survival <2 years (11.6 g/dL).

**Table 1:** Patient and disease characteristics of patients who received radium-223 in the real world.

Patient/disease characteristic		Survival			All patients^a^ (*N* = 1180)
		<6 months (*n* = 264)	<2 years (*n* = 775)^b^	≥2 years (*n* = 185)	
Age at start of radium-223, median (range), years		75.0 (53–85)	74.0 (44–85)	71.0 (47–83)	73.0 (44–85)
Race, *n* (%)					
White		203 (76.9)	589 (76.0)	132 (71.4)	867 (73.5)
Black/African American		19 (7.2)	51 (6.6)	13 (7.0)	81 (6.9)
Other^c^		29 (11.0)	87 (11.2)	34 (18.4)	155 (13.1)
Missing		13 (4.9)	48 (6.2)	6 (3.2)	77 (6.5)
ECOG performance status, *n* (%)					
0		38 (14.4)	115 (14.8)	34 (18.4)	195 (16.5)
1		86 (32.6)	226 (29.2)	45 (24.3)	338 (28.6)
2		52 (19.7)	102 (13.2)	6 (3.2)	119 (10.1)
3 or 4		10 (3.8)	16 (2.1)	1 (0.5)	20 (1.7)
Missing		78 (29.5)	316 (40.8)	99 (53.5)	508 (43.1)
Gleason score at diagnosis, *n* (%)					
>7		138 (52.3)	424 (54.7)	87 (47.0)	628 (53.2)
≤7		62 (23.5)	194 (25.0)	62 (33.5)	320 (27.1)
Unknown/not documented		64 (24.2)	157 (20.3)	36 (19.5)	232 (19.7)
Metastatic stage at diagnosis, *n* (%)					
M0		104 (39.4)	301 (38.8)	76 (41.1)	454 (38.5)
M1		109 (41.3)	292 (37.7)	62 (33.5)	453 (38.4)
Unknown/not documented		51 (19.3)	182 (23.5)	47 (25.4)	273 (23.1)
Site of metastasis at start of radium-223, *n* (%)					
Bone only		198 (75.0)	610 (78.7)	160 (86.5)	953 (80.8)
Distant lymph node ± bone		29 (11.0)	90 (11.6)	16 (8.6)	131 (11.1)
Visceral ± lymph node/bone		37 (14.0)	75 (9.7)	8 (4.3)	95 (8.1)
Missing		0	0	1 (0.5)	1 (<0.1)
Time from mCRPC to start of radium-223, months					
Mean (standard deviation)		12.9 (10.4)	12.7 (10.7)	10.6 (11.1)	12.9 (11.8)
Median (range)		10.6 (−3.9 to 59.1)	9.9 (−6.6 to 61.2)	7.1 (−8.1 to 54.8)	9.7 (−8.1 to 70.0)
Prior symptomatic skeletal events, *n* (%)		127 (48.1)	344 (44.4)	71 (38.4)	496 (42.0)
Laboratory values at baseline, median (range)					
Prior therapies before start of radium-223, *n* (%)^e^	Any	201	527	78	738
	Chemotherapy	108 (40.9)	250 (32.3)	30 (16.2)	337 (28.6)
	Novel hormone agents	191 (72.3)	493 (63.6)	60 (32.4)	679 (57.5)
	Bone health agents	176 (66.7)	492 (63.5)	84 (45.4)	689 (58.4)
Radium-223 line of therapy, *n* (%)^f^	1	53 (20.1)	202 (26.1)	87 (47.0)	357 (30.3)
	2	87 (33.0)	251 (32.4)	44 (23.8)	359 (30.4)
	3	58 (22.0)	159 (20.5)	26 (14.1)	226 (19.2)
	4	26 (9.8)	63 (8.1)	6 (3.2)	85 (7.2)
	5	17 (6.4)	33 (4.3)	2 (1.1)	44 (3.7)
	6	10 (3.8)	17 (2.2)	0	19 (1.6)
	7	3 (1.1)	4 (0.5)	0	5 (0.4)
	Missing	10 (3.8)	46 (5.9)	20 (10.8)	85 (7.2)

*ECOG* Eastern Cooperative Oncology Group, *M* metastasis, *mCRPC* metastatic castration-resistant prostate cancer.

^a^Full analysis set, including patients censored before 2 years.

^b^Includes the 264 patients with survival <6 months.

^c^Other race includes patients who identified as Asian, Hispanic, Latino, or “Other”.

^d^PSA value was set to 30,000 μg/L if the original value was larger than 30,000 μg/L.

^e^Patients could have received more than one treatment before radium-223 and could have continued to receive a therapy started before radium-223 initiation during and after they received radium-223.

^f^Includes use as monotherapy or in combination with life-prolonging therapies (e.g., abiraterone acetate and prednisone; enzalutamide; and/or taxanes) in the metastatic setting.

Differences in baseline characteristics were more pronounced between patients who survived ≥2 years versus <6 months. Lower proportions of men who survived ≥2 years versus <6 months had baseline Eastern Cooperative Oncology Group (ECOG) performance status 2–4 (4% vs 24%), documented visceral metastases (4% vs 14%), and prior history of SSEs (38% vs 48%), and baseline median serum ALP (78 U/L vs 168 U/L) and PSA levels (17 μg/L vs 133 μg/L) were lower (Table 1).

### Treatment exposure and sequencing

Radium-223 treatment exposure is shown in Table 2. In patients who survived ≥2 years versus <2 years, a higher proportion received radium-223 as a first-line treatment for mCRPC (monotherapy or combination; 47% vs 26%), and fewer patients received prior chemotherapy (16% vs 32%), prior novel hormone agents (i.e., abiraterone acetate and prednisone, or enzalutamide: 32% vs 64%), or prior bone health agents (e.g., denosumab or zoledronic acid; 45% vs 64%).

**Table 2:** Real-world use of radium-223 in patients with metastatic castration-resistant prostate cancer.

Patients treated with radium-223, *n* (%)	Survived <6 months (*n* = 264)	Survived <2 years (*n* = 775)^a^	Survived ≥2 years (*n* = 185)	All patients^b^ (*N* = 1180)
Monotherapy	162 (61.4)	446 (57.5)	86 (46.5)	654 (55.4)
Combination therapy	92 (34.8)	283 (36.5)	79 (42.7)	441 (37.4)
—Hormonal therapy	82 (31.1)	259 (33.4)	75 (40.5)	406 (34.4)
—Hormonal therapy + chemotherapy	5 (1.9)	12 (1.5)	1 (0.5)	15 (1.3)
—Chemotherapy	4 (1.5)	10 (1.3)	1 (0.5)	15 (1.3)
—Hormonal therapy + immunotherapy	0	1 (0.1)	1 (0.5)	2 (0.2)
—Other	1 (0.4)	1 (0.1)	1 (0.5)	3 (0.3)
Missing	10 (3.8)	46 (5.9)	20 (10.8)	85 (7.2)
Injections received^c^				
6	5 (1.9)	264 (34.1)	143 (77.3)	543 (46.0)
5 or 6	18 (6.8)	349 (45.0)	156 (84.3)	654 (55.4)
1–4	246 (93.2)	426 (55.0)	29 (15.7)	525 (44.5)

^a^Includes the 264 patients with survival <6 months.

^b^Full analysis set, including patients censored before 2 years.

^c^Radium-223 should be given as 6 injections at 4-week intervals; one patient censored within 2 years received 7 injections.

Among 738 patients who did not receive radium-223 as first-line therapy, most (92% overall) received prior novel hormone agents, while fewer than half (46%) received prior chemotherapy in the mCRPC setting. In the 78 patients who survived ≥2 years who did not receive radium-223 first line, 77% received prior novel hormone agents and 39% received prior chemotherapy for mCRPC. In the 527 patients who survived <2 years who did not receive radium-223 first line, 94% received prior novel hormone agents and 47% received prior chemotherapy for mCRPC.

Most patients (71%) who survived for ≥2 years received radium-223 as a first or second line of therapy; only 4% received radium-223 as a fourth or later line. In the group of patients who survived for <2 years, the proportion who received radium-223 in first or second line was lower (58%), while 15% of them received it as a fourth or later line. In patients who survived <6 months, the proportions were 53% and 21%, respectively. The proportion of patients who received radium-223 in combination with other life-prolonging therapies (most commonly novel hormone agents) was similar among patients who survived for ≥2 years (43%) or <2 years (37%).

Almost all patients (84%) who survived for ≥2 years received 5 or 6 of the planned 6 injections of radium-223, compared with 45% of patients who survived for <2 years. Reasons for the reduced number of injections were not captured; however, it should be noted that 34% of patients (264 of 775) in the <2-year survival group survived for <6 months; therefore, many patients would not have had the opportunity to complete 5 or 6 months of treatment.

### Overall survival

**Fig. 2:**
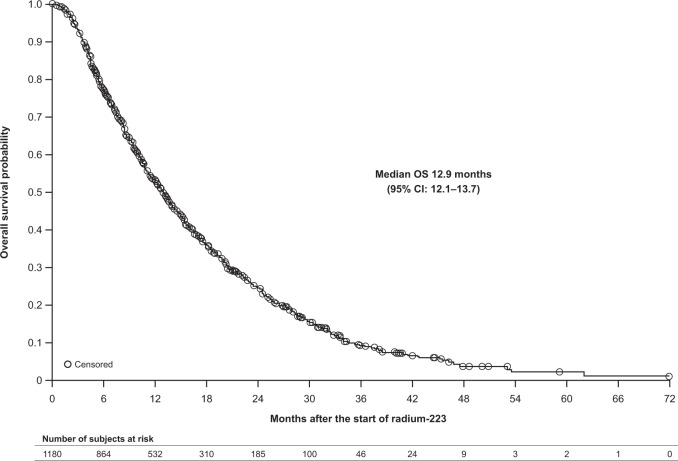
Overall survival. Kaplan–Meier estimate; full-analysis set.

Median OS from the start of radium-223 was 12.9 months (95% CI: 12.1–13.7) (Fig. 2). On univariate analysis, factors significantly associated with reduced OS were age >75 versus <65 years, baseline ECOG performance status 2–4 versus 0, presence of visceral versus bone-only metastases, raised baseline levels of ALP, LDH, and PSA, prior SSEs, prior chemotherapy, and prior therapy with a bone health agent; whereas “other” race (including Asian, Hispanic, Latino, and “other”, as self-identified by the patient) and higher baseline hemoglobin levels were significantly associated with improved OS (Supplementary Table S1). On multivariate analysis, age >75 years, ECOG performance status 2–4 at baseline, presence of visceral metastases, prior SSEs, and prior chemotherapy were independently prognostic of reduced OS, whereas “other” race was independently prognostic of improved OS (Table 3).

**Table 3:** Baseline patient and disease characteristics independently prognostic of overall survival in standard multivariate analysis (full-analysis set)^a^.

Characteristic	Variable class	Hazard ratio	95% CI	*p* value
Age at start of radium-223 (reference: <65 years)	65–75 years	1.08	0.90–1.31	0.4183
	>75 years	1.46	1.21–1.77	<0.0001
Race (reference: white)	Black or African American	0.95	0.73–1.24	0.7217
	Missing	1.20	0.90–1.59	0.2126
	Other^b^	0.68	0.55–0.84	0.0003
Baseline ECOG performance status (reference: 0)	1	1.15	0.93–1.41	0.2033
	2–4	2.03	1.58–2.60	<0.0001
	Missing	1.05	0.86–1.28	0.6428
Site of metastasis (reference: bone only)^c^	Distant lymph node ± bone	1.18	0.95–1.45	0.1329
	Visceral ± lymph node/bone	1.61	1.27–2.03	<0.0001
Prior symptomatic skeletal event (reference: no)	Yes	1.19	1.04–1.37	0.0096
Prior chemotherapy (reference: no)	Yes	1.57	1.35–1.84	<0.0001

*CI* confidence interval, *ECOG* Eastern Cooperative Oncology Group.

^a^The initial model included age at start of radium-223, race, practice type, Gleason score at diagnosis, region, histology, group stage, tumor (T) stage, node (N) stage, metastasis (M) stage, baseline ECOG performance status, site of metastasis, prior bone health agent, prior symptomatic skeletal event, and prior chemotherapy. Baseline laboratory variables were not included in the model selection process, because of the high proportion of missing values.

^b^Other race includes patients who identified as Asian, Hispanic, Latino, or “Other”.

^c^One patient with missing site of metastasis was removed from the analysis.

### PSA and ALP responses

Within 6 months after initiation of radium-223 therapy (either as monotherapy or in combination with another life-prolonging therapy), a reduction in PSA level of ≥50% from baseline was observed in 74 (13%) of 566 patients with baseline PSA ≥10 μg/L and ≥1 PSA measurement post-baseline.

Among patients with a baseline ALP measurement and at least one measurement >15 days after starting radium-223, an ALP response (≥30% ALP reduction from baseline) was achieved in 387 of 849 patients (46%) overall, 68 of 107 patients (64%) who survived ≥2 years, 247 of 584 patients (42%) who survived <2 years, and 57 of 220 patients (26%) who survived <6 months.

## Discussion

In this analysis of real-world radium-223 use, the median OS was slightly shorter than that seen in the ALSYMPCA trial (12.9 vs 14.9 months [1]), which was conducted in an era before the availability of abiraterone and enzalutamide. In current clinical practice, several treatment options are available to patients, and our analysis shows that radium-223 is used in various treatment lines and in combination with other life-prolonging anticancer therapies in a high proportion of patients (37% overall). The shorter median OS in comparison with ALSYMPCA suggests that radium-223 is being used in a wider population, including those with poorer prognostic features such as the presence of visceral metastases, which were an exclusion criterion in ALSYMPCA. According to its licensed indication, radium-223 should not be used in patients with visceral metastasis. In this retrospective review of patient health records, detailed insights into the treatment decisions for each patient with visceral metastases are not available, but we can assume that the treating physicians would have considered the expected benefit for individual patients. It cannot be excluded that patients with visceral metastases received combination treatment with other agents or may have had stable visceral metastases with symptomatic bone metastasis. Slightly more patients with survival <2 years received chemotherapy as concomitant therapy, compared with patients who survived ≥2 years. The low PSA response rate (13%) in this analysis reflects the mechanism of action of radium-223, which is bone targeted and has no direct impact on the androgen receptor signaling pathway. Of note, previous analyses have shown that neither PSA response nor PSA progression at week 12 is a prognostic marker of survival in men with CRPC and symptomatic bone metastases [6]. An ALP response (30% reduction from baseline at any time after radium-223 initiation) was more common in patients with survival ≥2 years versus <2 years.

We were interested in characterizing patients with longer versus shorter survival following radium-223 therapy, and we prespecified 2 years as the minimum for clinically relevant “long-term” survival. When we subsequently analyzed the OS data in quartiles, the cut-offs for the shortest and longest quartiles were ~6 months and 2 years, supporting our categories. Given the small group sizes, we did not undertake statistical comparisons of the survival groups. However, on a purely descriptive analysis, we saw better ECOG performance status, bone-only metastases, a slightly lower incidence of prior SSEs, less use of prior chemotherapy, lower baseline levels of ALP and LDH, and greater hemoglobin levels in patients with survival ≥2 years versus survival <2 years. Furthermore, proportionally more patients who survived ≥2 years versus <2 years were treated with radium-223 as their first or second line of therapy (71% vs 58%) and received 5–6 injections of radium-223 (84% vs 45%). Many of these characteristics are known favorable prognostic factors associated with longer survival in patients with mCRPC [7–10], whereas other unfavorable prognostic factors were not associated with worse outcomes in our analysis. Taken together, these findings seem to support the survival benefit of early treatment initiation in patients with good prognostic factors. These results also indicate that radium-223, as appropriate, could be considered as one of the early treatment options integrated into the management of patients with mCRPC, with no apparent adverse impact on long-term prognosis.

Other real-world studies have shown correlations between overall survival and completion of 5 or 6 radium-223 injections, use of radium-223 in the first or second line, or use before chemotherapy [11–18]. Patients able to complete the full course of radium-223 injections are likely to have fewer symptoms (including less pain), less disease burden, a good performance status, low baseline PSA and ALP levels, and high baseline hemoglobin levels [11, 19–23]. Although these findings from real-world studies require confirmation in a prospectively designed clinical trial, they support our belief that initiation of radium-223 early in the mCRPC disease course is appropriate, while patients have a lower burden of disease and are still fit enough to receive the full treatment course. Such use of radium-223 could avoid the drawbacks of sequential use of novel hormone agents, which may have limited and short-lived benefit [24–30], while acknowledging physicians’ and patients’ preferences to delay chemotherapy to avoid toxicities that affect quality of life [2, 31–33]. Consideration of the overall place of radium-223 in treatment sequencing is now particularly important given the increasing number of therapies available, and the opportunity for patients to receive more lines of therapy and to start other systemic therapies at an earlier disease stage [34]. Indeed, with the growing use of other systemic treatments in the metastatic hormone-sensitive prostate cancer space, there could be a role for radium-223 at the time of development of castration resistance in patients whose disease is already resistant to novel hormonal agents and/or docetaxel chemotherapy.

Of specific importance to treatment sequencing considerations is that radium-223 is well tolerated, and chemotherapy can be administered after radium-223 without excess hematologic toxicity [1, 3, 35–38]. Whereas the benefit to be gained when radium-223 is used after chemotherapy appears to be limited, radium-223 can also be used before or after novel hormone agents [18, 39–42]. In the ERA 223 trial, an increased fracture incidence with radium-223 in combination with abiraterone versus abiraterone alone was reported; however, in the PEACE III trial, this risk was largely mitigated when bone health agents were added to the combination of radium-223 plus enzalutamide versus enzalutamide alone [43–45]. Ideally, radium-223 should be initiated in bone-dominant disease when initial therapy is no longer effective [4, 46], and important factors to contemplate when considering radium-223 treatment are progressive bone metastases, emerging or progressive symptoms, limited or stable lymph node disease, and the absence of visceral metastases [1, 4, 12, 47]. Radium-223 is administered as 6 injections at 4-week intervals, and thus can be completed relatively quickly, allowing patients to move on to subsequent therapies to maximize OS. Nevertheless, absolute clarity on treatment sequencing remains elusive.

A weakness of this study is that it was a retrospective analysis and therefore has the potential for selection bias. In addition, missing data at baseline and other timepoints—a problem common to real-world analyses—resulted in censoring of a number of patients. Nevertheless, strengths of real-world data are that they more accurately represent the population of patients seen in clinical practice and further define long-term safety and efficacy in a more diverse population than is included in clinical trials. These data therefore complement the ALSYMPCA findings.

## Conclusions

This retrospective study of patients with mCRPC confirms the prognostic role of lower disease burden and bone-only disease. In addition, we found that proportionally more patients who survived ≥2 years versus <2 years were treated with radium-223 as first- or second-line treatment and were able to complete 5 or 6 cycles of radium-223. We therefore suggest that, for selected patients, starting radium-223 early in the mCRPC disease course, while they are still relatively fit and able to receive the full 6 injections, may be an appropriate treatment strategy to help improve clinical outcomes.

## Supplementary information

Supplementary information

## Data Availability

The data underlying this publication were provided by Flatiron under contract to Bayer. Requests for access to the data should be made to Flatiron (https://flatiron.com).
