# Supplementary material for: Real-world patient characteristics associated with survival of 2 years or more after radium-223 treatment for metastatic castration-resistant prostate cancer (EPIX study)
Source: Prostate Cancer Prostatic Dis. 2022 Feb 21;25(2):306–13. doi: 10.1038/s41391-021-00488-0 (PMC9184267; doi:10.1038/s41391-021-00488-0)
Supplement: Supplementary file 1 — Supplementary information [file 41391_2021_488_MOESM1_ESM.docx]

**Real-world patient characteristics associated with prolonged survival after radium-223 treatment for metastatic castration-resistant prostate cancer (EPIX study)**

Daniel J. George, Neeraj Agarwal, Oliver Sartor, Cora N. Sternberg, Bertrand Tombal, Fred Saad, Kurt Miller, Niculae Constantinovici, Helen Guo, John Reeves, XiaoLong Jiao, Per Sandström, Frank Verholen, Celestia S. Higano, Neal Shore

**Supplementary Table S1** Overall survival: standard univariate Cox proportional hazards models (full-analysis set).^a^

| **Characteristics** | **Hazard ratio** | **95% CI** | **P value** |
| --- | --- | --- | --- |
| Age at start of radium-223 (reference: <65 years) |  |  |  |
| 65–75 years | 0.99 | 0.82–1.18 | 0.8732 |
| >75 years | 1.32 | 1.10–1.58 | 0.0031 |
| Race (reference: white) |  |  |  |
| Black or African American | 0.97 | 0.74–1.26 | 0.8033 |
| Missing | 1.09 | 0.82–1.44 | 0.5619 |
| Other^b^ | 0.69 | 0.56–0.85 | 0.0006 |
| Baseline ECOG performance status (reference 0) |  |  |  |
| 1 | 1.22 | 0.99–1.51 | 0.0560 |
| 2–4 | 2.17 | 1.70–2.77 | <0.0001 |
| Missing | 1.00 | 0.82–1.22 | 0.9852 |
| Site of metastasis (reference: bone only) |  |  |  |
| Distant lymph node ± bone | 1.23 | 1.00–1.52 | 0.0499 |
| Visceral ± lymph node/bone | 1.74 | 1.38–2.19 | <0.0001 |
| Log-transformed baseline alkaline phosphatase, U/L | 1.60 | 1.45–1.75 | <0.0001 |
| Baseline hemoglobin, g/dL | 0.75 | 0.71–0.79 | <0.0001 |
| Log-transformed baseline lactate dehydrogenase, U/L | 1.85 | 1.41–2.42 | <0.0001 |
| Log-transformed baseline prostate-specific antigen, μg/L^c^ | 1.23 | 1.19–1.28 | <0.0001 |
| Prior symptomatic skeletal event (reference: no) |  |  |  |
| Yes | 1.23 | 1.08–1.40 | 0.0024 |
| Prior chemotherapy (reference: no) |  |  |  |
| Yes | 1.59 | 1.37–1.83 | <0.0001 |
| Prior bone health agent (reference: no) |  |  |  |
| Yes | 1.41 | 1.23–1.61 | <0.0001 |

^a^ Univariate models of site of metastasis, baseline alkaline phosphatase (U/L), baseline hemoglobin (g/dL), baseline lactate dehydrogenase (U/L), baseline prostate-specific antigen (μg/L) only include non-missing values.

^b^ Other race includes patients who identified as Asian, Hispanic, Latino, or “Other”.

^c^ Prostate-specific antigen is set to 30 000 μg/L if the original value is >30 000 μg/L.

*CI* confidence interval; *ECOG* Eastern Cooperative Oncology Group.
